# Internet and Media Use in the Context of Infertility and Assisted Reproductive Technology: Empowerment or Overload?

**DOI:** 10.7759/cureus.107689

**Published:** 2026-04-25

**Authors:** Imen Bannour, Ekram Guerbej, Mariem Romdhani, Rania Bannour, Hafedh Abbassi, Soumaya Dali, Sihem Chahed, Sassi Boughizane

**Affiliations:** 1 Department of Obstetrics and Gynecology, Faculty of Medicine of Sousse/Farhat Hached University Hospital, Sousse, TUN; 2 Department of Community Medicine, LR12ES03, Faculty of Medicine Ibn El Jazzar of Sousse, University of Sousse, Sousse, TUN; 3 Department of Obstetrics and Gynecology, Higher School of Health Sciences and Techniques of Monastir, University of Monastir, Monastir, TUN

**Keywords:** assisted reproductive technologies (art), effects of social media, infertility, information quality, internet technologies

## Abstract

Background

Infertility is a major public health issue affecting approximately one in six couples worldwide. Beyond its medical implications, it carries significant psychological and social burdens, particularly in contexts where procreation is highly valued. Faced with barriers to accessing assisted reproductive technology (ART), many women turn to the Internet and media as primary sources of information. However, the reliability and clarity of online content remain variable, sometimes contributing to misinformation and increased anxiety.

Objective

To assess the frequency, sources, motivations, trust, satisfaction, and perceived impact of Internet and media use for infertility and ART-related information among women consulting in a specialized reproductive health center.

Methods

We conducted a prospective observational descriptive cross-sectional study between January and March 2025 among 100 women of reproductive age consulting for infertility in a specialized center. Participants were recruited randomly and completed a self-administered questionnaire exploring sociodemographic characteristics, digital practices, information sources, level of trust, satisfaction, and perceived impact. Data were analyzed using Epi Info™ version 7.2.5.0 (CDC, Atlanta, GA, USA).

Results

Almost all participants reported using the Internet for infertility or assisted reproductive technology-related information (99/100, 99%). The most frequently consulted sources were specialized medical websites (57/100, 57%), social media platforms (56/100, 56%), and online videos (53/100, 53%). The most commonly searched topics were treatment options (79/100, 79%), success rates (70/100, 70%), and causes of infertility (67/100, 67%). The main motivations for Internet use were better understanding of the condition (78/100, 78%), exploring therapeutic options (63/100, 63%), and obtaining a second opinion (40/100, 40%). Online information improved knowledge for 85 participants (85/100, 85%) and facilitated decision-making for 78 participants (78/100, 78%). However, increased stress related to online information was reported by 80 participants (80/100, 80%), and 34 participants (34/100, 34%) felt that the available information was not adapted to their personal situation. Despite this, high satisfaction with information provided by healthcare professionals was reported by 88 participants (88/100, 88%).

Conclusion

The Internet and media have become indispensable sources of information for women facing infertility, supporting knowledge acquisition and decision-making. However, the heterogeneity and potential inaccuracy of online resources may increase patient anxiety. These findings highlight the importance of healthcare professionals in guiding patients toward reliable, evidence-based, and contextualized information to optimize infertility care.

## Introduction

Infertility, defined as the inability to achieve pregnancy after 12 months or more of regular unprotected sexual intercourse, represents a major global public health concern, affecting approximately 17.5% of the adult population worldwide [1,2]. Its prevalence is similar in high-income countries (17.8%) and in low- and middle-income countries (16.5%), though regional disparities exist, with rates ranging from 10.7% in the Middle East and North Africa to 23.2% in the Western Pacific region [3]. In many settings, particularly in Africa, infertility is not only a medical condition but also a significant social and psychological burden, often leading to stigmatization, marginalization, and impaired quality of life for women [4,5].

Assisted reproductive technology (ART), despite being a major biomedical advancement, remains inaccessible to a large proportion of infertile couples due to high costs, prolonged treatment duration, and substantial emotional burden, with no guarantee of success [6,7]. Confronted with these barriers, many women increasingly turn to the Internet and media to obtain medical information, seek peer experiences, or explore treatment alternatives. However, the quality, reliability, and readability of online content vary considerably, exposing patients to the risk of misinformation [8].

In this context, healthcare professionals play an essential role in directing infertile couples toward trustworthy and evidence-based resources [9]. The present cross-sectional descriptive study was conducted to describe the frequency and modalities of Internet and media use for infertility-related information among women consulting in a specialized ART center and to explore their motivations and self-reported perceptions regarding the reliability and perceived effects of such information.

## Materials and methods

Study period

Data collection was carried out over a three-month period, from January to March 2025.

Eligibility criteria

**Figure 1:**
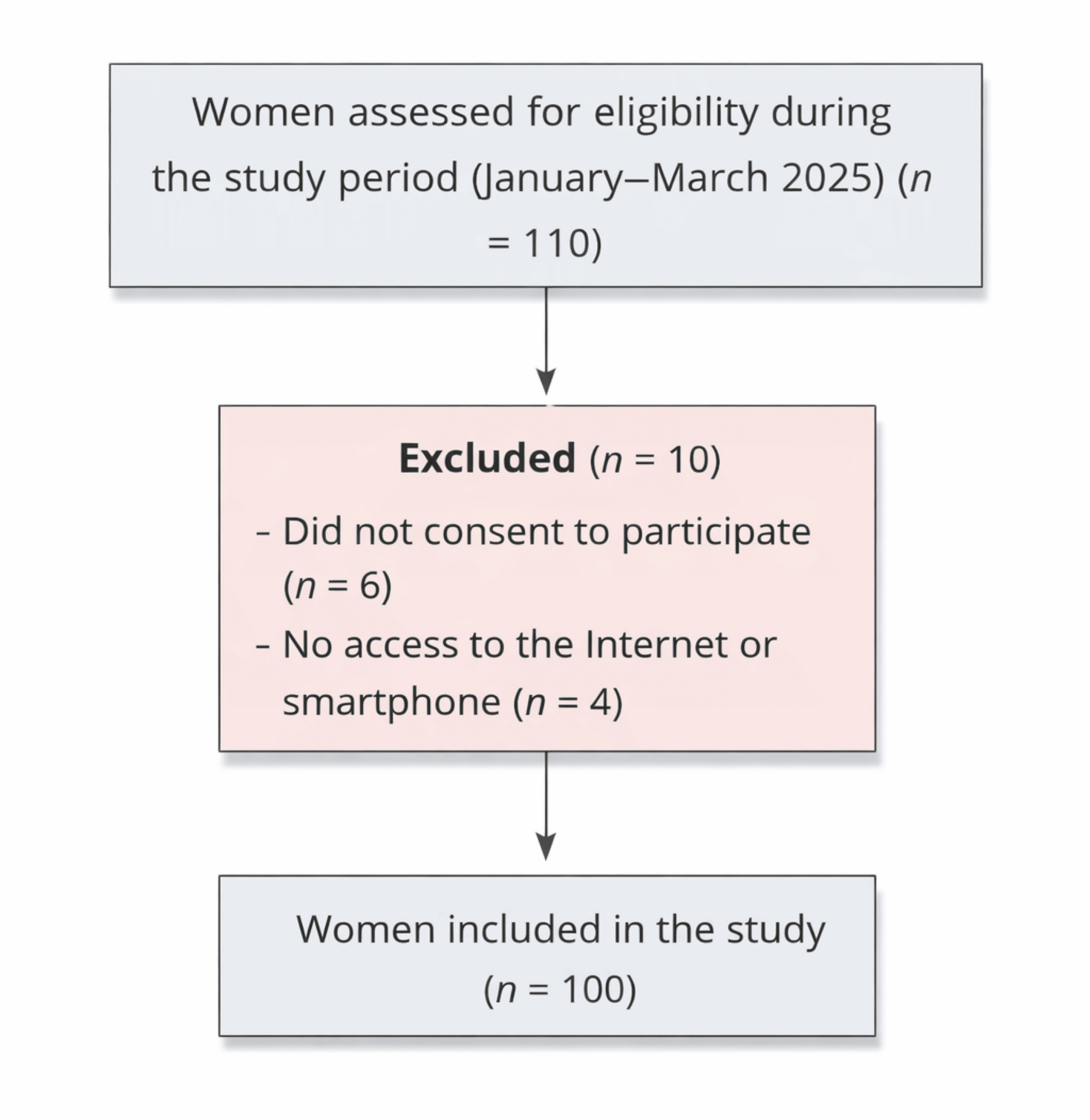
Flowchart of participant selection for the study

Inclusion criteria comprised all women of reproductive age consulting for infertility who agreed to participate in the study. Women who did not consent to participate or who were unable to use the Internet or did not have access to a smartphone were excluded. Women with previous psychiatric diagnoses, a history of infertility treatments (including ovulation induction, intrauterine insemination, or assisted reproductive technology), or previous pregnancy loss were not excluded, as these characteristics are common among infertile populations and were considered part of the clinical heterogeneity of the study sample. A flowchart illustrating participant inclusion and exclusion criteria is provided in Figure 1.

Data collection tools and procedures

Data were collected using a structured self-administered questionnaire specifically developed for this study. The questionnaire was administered at the time of consultation in the specialized infertility center, regardless of the stage of the infertility care pathway (initial consultation, ongoing treatment, or after one or more failed attempts). This approach was intended to reflect real-life variability in patients’ experiences and information-seeking behaviors across different stages of infertility management.

The instrument included 32 items divided into five domains: sociodemographic characteristics, patterns of Internet and media use, motivations for online searches, role of health professionals, and perceived impact and satisfaction. To enhance construct validity, items related to “Level of Trust” and “Perceived Impact” were developed based on a review of existing literature on health information-seeking behavior and patient-reported outcomes in infertility and digital health contexts. These items were designed using simple, clearly defined Likert-type scales to capture subjective perceptions consistently. Additionally, the questionnaire was pretested among 10 women of reproductive age to assess clarity, relevance, and comprehension, ensuring that the constructs were appropriately understood by participants. The level of trust in online information was assessed using a self-reported Likert-type scale with predefined response categories (“no trust,” “moderate trust,” “high trust,” and “absolute trust”). These categories reflected participants’ subjective perception of trust and were not based on a numerical scoring system or predefined cutoff values.

Most items were closed-ended and used multiple-choice or Likert-type scales. The questionnaire was pretested among 10 women of reproductive age, not included in the final analysis, to ensure clarity and content validity. No modifications were necessary after pretesting.

Data management and statistical analysis

Collected data were coded and entered into a predesigned data management grid and analyzed using Epi Info™ version 7.2.5.0 (CDC, Atlanta, GA, USA). Only descriptive statistical analysis was performed. Categorical variables were summarized as absolute frequencies and percentages, and continuous variables were described using means and standard deviations.

Given the exploratory nature and primary descriptive objective of the study, no inferential or bivariate analyses were conducted, as the study was not specifically powered to detect statistically significant associations between variables.

Regarding data completeness, all returned questionnaires were reviewed prior to analysis. Questionnaires with substantial missing data were excluded, while partially completed questionnaires with minimal missing responses were retained and analyzed based on available data. The proportion of missing data was low and did not affect the overall analysis.

Ethical considerations

All participants provided informed consent prior to completing the questionnaire, which was considered as implied consent upon voluntary participation. Ethical approval for this study was obtained from the local Institutional Review Board (IRB) (approval number: CEFMSo_0178_2025).

## Results

The study population consisted of 100 infertile women attending the ART center. The mean age was 32.9 ± 5.5 years (range: 22-44). The most represented age group was 25-35 years (51/100, 51%), followed by 35-50 years (41/100, 41%) and younger than 25 years (8/100, 8%). More than half of the respondents had a secondary level of education (57/100, 57%), 36 participants had a university education (36/100, 36%), and seven had only primary education (7/100, 7%). Socioeconomic status (SES) was predominantly middle (83/100, 83%), with 10 participants reporting low SES (10/100, 10%) and seven reporting high SES (7/100, 7%). The majority resided in urban areas (80/100, 80%), while 20 lived in rural settings (20/100, 20%). Most participants were nulligravid (79/100, 79%), 16 were primigravid (16/100, 16%), and five were multigravid (5/100, 5%). Nulliparity was reported in 90 participants (90/100, 90%). Accordingly, 90 presented with primary infertility (90/100, 90%) and 10 with secondary infertility (10/100, 10%). The mean duration of infertility was 72.6 ± 44.5 months (range: 18-204 months) (Table 1).

**Table 1:** Sociodemographic and Clinical Characteristics of the Patients (n=100) *Patients could have received more than one infertility treatment modality; therefore, percentages may exceed 100%.

Characteristics	Subcategories	Number of patients (n)	Percentage (%)
Age group	< 25 years	8	8
	25–35 years	51	51
	35–50 years	41	41
Educational level	Primary	7	7
	Secondary	57	57
	University	36	36
Socioeconomic status	Low	10	10
	Middle	83	83
	High	7	7
Place of residence	Urban	80	80
	Rural	20	20
Gravidity	Nulligravid	79	79
	Primigravid	16	16
	Multigravid	5	5
Parity	Nulliparous	90	90
	Primiparous	10	10
History of miscarriage	None	87	87
	One	9	9
	Two or more	4	4
Type of infertility	Primary infertility	90	90
	Secondary infertility	10	10
Infertility treatment*	Ovulation induction (OI)	40	40
	Intrauterine insemination (IUI)	40	40
	In vitro fertilization (IVF)	34	34
	Intracytoplasmic sperm injection (ICSI)	13	13

**Figure 2:**
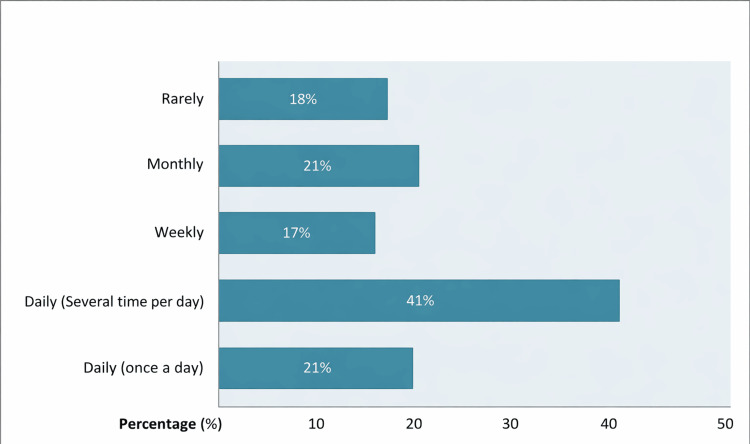
Distribution of Participants According to Their Frequency of Internet Use for Infertility-Related Searches (n = 100)

Nearly all participants reported using the Internet to seek information on infertility or assisted reproductive technology (99/100, 99%). More than two-thirds had been using the Internet for infertility-related information for over one year (71/100, 71%), and 41 participants reported searching online several times per day (41/100, 41%) (Figure 2).

Specialized medical websites were the most frequently consulted sources (57/100, 57%), followed by social media platforms (56/100, 56%) and online videos such as YouTube (53/100, 53%). Only three participants reported using forums or support groups (3/100, 3%). Trust in online information was reported as moderate by 53 participants (53/100, 53%), while 34 expressed full confidence (34/100, 34%) and three reported no trust at all (3/100, 3%). A majority indicated that their confidence was reinforced when information was corroborated by testimonies from other patients (60/100, 60%), while 43 participants valued information from reputable sources such as hospitals, clinics, and experts (43/100, 43%). Furthermore, 60 participants first used the Internet before undergoing their initial attempt at simple induction or assisted reproductive technology procedures (60/100, 60%). Treatment options were the most frequently sought type of information (79/100, 79%), followed by success rates (70/100, 70%) and causes of infertility (67/100, 67%). More than three-quarters reported using the Internet to better understand their condition (78/100, 78%), 63 to explore treatment options (63/100, 63%), and 47 to read patient testimonies (47/100, 47%). About two-thirds cited the availability of peer experiences as an advantage of online resources (63/100, 63%), while 48 highlighted the speed and ease of access (48/100, 48%), and 28 appreciated the diversity of sources (28/100, 28%) (Table 2).

**Table 2:** Distribution of Patients According to Internet and Media Use for Infertility and ART Information (n = 100) Multiple responses were allowed for several items; therefore, percentages may exceed 100%. * Trust levels were self-reported using predefined Likert-type categories and did not rely on numerical score thresholds.

Characteristics	Item	Number (n)	Percentage (%)
Most frequently consulted online sources	Specialized medical websites	57	57
	YouTube channels / online videos	53	53
	Forums and support groups	3	3
	Social media (Facebook, Instagram, etc.)	56	56
	All sources	1	1
	None	1	1
Level of trust in online information about infertility*	No trust	3	3
	Moderate trust (some uncertainty)	10	10
	High trust (rather confident)	53	53
	Absolute trust (very confident)	34	34
Reasons for trust or mistrust	Information from reputable sites (hospitals, clinics, experts)	43	43
	Concordant patient testimonies	60	60
	Lack of source verification	19	19
	Contradictory or unclear content	7	7
Timing of first Internet use for infertility/ART	Before the first induction or ART attempt	63	63
	After the first failed attempt	23	23
	After several failed attempts	13	13
	Never	1	1
Types of information searched online	Causes of infertility	67	67
	Treatment options (ART, IVF, etc.)	79	79
	Patient testimonies and experiences	47	47
	Support groups / online forums	2	2
	Treatment success rates	70	70
Perceived advantages of Internet use	Speed and ease of access	48	48
	Diversity of sources	28	28
	Availability of patient testimonies	63	63
	Contact and discussion with peers in similar situations	31	31

One-third of the women reported searching online for information prior to their first fertility consultation (33/100, 33%). A majority indicated that online resources helped them prepare for medical appointments (78/100, 78%). Additionally, 94 participants reported using the Internet to improve understanding of their condition (94/100, 94%), 83 to obtain a second opinion (83/100, 83%), and 68 to seek emotional support (68/100, 68%). While 85 participants acknowledged improved knowledge (85/100, 85%) and 78 reported facilitated decision-making (78/100, 78%) following online information use, 80 identified increased anxiety or stress as a major drawback (80/100, 80%). Furthermore, 34 stated that available information lacked adaptation to their specific situation (34/100, 34%). Nearly half of the respondents reported moderate satisfaction with online information (46/100, 46%), whereas 34 were highly satisfied (34/100, 34%) and 14 were minimally satisfied (14/100, 14%). In contrast, the vast majority reported being very satisfied with information provided by healthcare professionals (88/100, 88%) (Table 3 and Table 4).

**Table 3:** Distribution of Patients According to Their Motivations for Using Media and the Internet for Fertility-Related Information

Characteristics	Response	Number (n)	Percentage (%)
Motivations for Internet use	Desire to better understand the fertility problem (Yes)	94	94
	Desire to better understand the fertility problem (Neutral)	3	3
	Desire to better understand the fertility problem (No)	3	3
	Seeking emotional support (Yes)	68	68
	Seeking emotional support (Neutral)	7	7
	Seeking emotional support (No)	25	25
	Obtaining a second opinion (Yes)	83	83
	Obtaining a second opinion (Neutral)	9	9
	Obtaining a second opinion (No)	8	8
	Dissatisfaction with information provided by healthcare professionals (Yes)	7	7
	Dissatisfaction with information provided by healthcare professionals (Neutral)	6	6
	Dissatisfaction with information provided by healthcare professionals (No)	87	87
Consequences of Internet use	Knowledge about fertility problems improved (Yes)	85	85
	Knowledge about fertility problems improved (Neutral)	9	9
	Knowledge about fertility problems improved (No)	6	6
	Decision-making regarding treatment was facilitated (Yes)	78	78
	Decision-making regarding treatment was facilitated (Neutral)	15	15
	Decision-making regarding treatment was facilitated (No)	7	7
	Discussion of Internet information with healthcare professionals (Yes)	31	31
	Discussion of Internet information with healthcare professionals (Neutral)	7	7
	Discussion of Internet information with healthcare professionals (No)	62	62
Information characteristics	Information was easy to find (Yes)	91	91
	Information was easy to find (Neutral)	7	7
	Information was easy to find (No)	2	2
	Information was accurate and reliable (Yes)	71	71
	Information was accurate and reliable (Neutral)	20	20
	Information was accurate and reliable (No)	9	9
	Information was confusing and difficult to understand (Yes)	13	13
	Information was confusing and difficult to understand (Neutral)	16	16
	Information was confusing and difficult to understand (No)	71	71

**Table 4:** Distribution of Participants According to Their Satisfaction with Information Provided Online and by Healthcare Professionals

Characteristics	Level of satisfaction	Number (n)	Percentage (%)
Satisfaction with online information	Very satisfied	34	34
	Moderately satisfied	46	46
	Slightly satisfied	14	14
	Not satisfied	6	6
Satisfaction with information provided by healthcare professionals	Very satisfied	88	88
	Moderately satisfied	7	7
	Slightly satisfied	2	2
	Not satisfied	3	3

## Discussion

According to the World Health Organization (WHO), infertility is an increasingly prevalent public health issue, affecting approximately one in six adults worldwide [1]. In our study, 90 participants reported primary infertility (90/100, 90%), while 10 presented with secondary infertility (10/100, 10%). These findings are consistent with a 2005 UK study involving 106 infertile patients, which reported 65% primary and 35% secondary infertility cases [10].

Our results show that nearly all respondents used the Internet to obtain information regarding infertility or assisted reproductive technology (99/100, 99%). This aligns with a Canadian study in which 87.8% of 558 participants reported using the Internet for fertility-related health information [11]. Similarly, a recent survey conducted in the United States indicated that 81.15% of the general population seeks health information online, with higher usage reported among younger individuals, non-Hispanic white populations, those with higher education and income levels, and urban residents [12].

In our cohort, 41 participants reported using the Internet multiple times daily to research infertility (41/100, 41%), whereas a 2003 Dutch study found that the majority of 72 couples (67%) accessed the Internet only once a month or less for fertility-related information [8]. A Turkish study conducted in 2016 similarly showed that 51.7% of infertile women used the Internet for infertility-related purposes one to two times per month [13]. These observations suggest a temporal evolution in digital health-seeking behaviors, with contemporary patients increasingly relying on online resources.

Specialized medical websites (57/100, 57%) and social media platforms (56/100, 56%) were the primary sources consulted in our study, whereas a Brazilian study published in 2024 highlighted a predominant reliance on Instagram and other social networks for infertility-related information [14]. These findings underscore the growing role of digital platforms in disseminating health information, while also drawing attention to concerns regarding variable content quality and potential exposure to misinformation [15,16]. In our cohort, increased stress related to online searches was reported by 80 participants (80/100, 80%). Previous studies have reported similar experiences, noting that online infertility forums may expose patients to negative narratives, misleading content, and emotionally distressing information [17]. Furthermore, difficulties in evaluating or filtering online information may contribute to cognitive overload and psychological distress [18,19].

An apparent paradox emerges from our findings: although a majority of participants reported that online information was easy to access (91%) and generally reliable (71%), a high proportion (80%) also reported increased anxiety associated with its use. This observation may be explained by several mechanisms.

First, even when information is factually accurate, exposure to extensive, detailed, or probabilistic medical data - such as treatment failure rates, complications, or uncertain outcomes - may heighten emotional distress, particularly in a vulnerable population already experiencing infertility-related stress. Second, the process of repeated online searching itself, often referred to as “cyberchondria,” may amplify anxiety through continuous exposure to conflicting or negatively framed information.

Furthermore, the lack of personalization of online content may contribute to this phenomenon, as patients may struggle to contextualize general medical information to their individual situation. These findings highlight that the impact of online health information is not solely determined by its accuracy but also by how it is interpreted, processed, and emotionally experienced by patients.

Our results also indicate that treatment options (79/100, 79%) and success rates (70/100, 70%) were the most frequently searched topics online, consistent with the findings of Brochu et al. (2019), who observed that infertile couples primarily seek information related to medical treatments and success outcomes [11]. This reflects a dual need for scientific information and experiential knowledge from peers, highlighting the perceived importance of online communities for emotional support [20,21]. The role of peer testimonies appears particularly relevant, as 63 participants perceived them as a benefit of online research (63/100, 63%). Previous studies have shown that women, more than men, actively engage in online support groups to share experiences and reduce feelings of isolation during their infertility journey [13,22].

Despite the widespread use of digital platforms, only 10 participants reported using the Internet due to a perceived lack of information from healthcare professionals (10/100, 10%). This contrasts with some European studies in which patients frequently report dissatisfaction with the adequacy of medical information received [23]. Nevertheless, our findings emphasize the central role of healthcare providers in patient education and support, as 88 participants reported high satisfaction with the information provided by their physicians (88/100, 88%).

Interestingly, despite this high level of satisfaction, only a minority of participants reported discussing information obtained online with their healthcare providers. Although this study did not specifically investigate the underlying reasons for this communication gap, several hypotheses may be considered. Patients may perceive online information-seeking as a personal or private activity and may hesitate to disclose it due to fear of being judged, dismissed, or not taken seriously by clinicians. Additionally, time constraints during consultations and the hierarchical nature of the physician-patient relationship in certain cultural contexts may further limit open discussion. To bridge this gap, clinicians should proactively inquire about patients’ use of online resources, encourage open dialogue, and adopt a non-judgmental approach. Integrating discussions about online information into routine consultations may help align patient perceptions with medical guidance, reduce misinformation-related anxiety, and strengthen the therapeutic relationship. International guidelines, such as those of the European Society of Human Reproduction and Embryology (ESHRE), emphasize the importance of continuous, person-centered communication to reduce anxiety and optimize treatment outcomes [24].

Strengths and limitations

This study has several strengths, including its systematic cross-sectional design, the use of random sampling, and a relatively large sample size of 100 participants, which enhanced the validity and representativeness of the findings. The structured, self-administered questionnaire was specifically developed for this research, covered multiple domains (sociodemographic characteristics, online practices, motivations, the role of healthcare professionals, and perceived impact), and was piloted to ensure clarity and feasibility. The high response rate further minimized the risk of non-response bias. However, the study also has limitations. The cross-sectional design precludes causal inference, and reliance on self-reported data may have introduced recall or social desirability bias.

Conducting the survey in a single specialized center may limit generalizability, and the questionnaire, though pretested, was not formally validated against international standards, which may affect cross-study comparability. In addition, as the questionnaire was administered within a clinical setting, responses may have been influenced by social desirability bias. Participants may have tended to underreport distrust in healthcare providers or overreport satisfaction, particularly in a context where care is actively being provided. This potential bias should be considered when interpreting the findings related to patient satisfaction and trust.

Future research should investigate qualitative aspects of online health-seeking behaviors, explore the psychological mechanisms underlying stress, and employ multi-center or longitudinal designs. Interventions such as physician-guided digital resources, patient education workshops, and digital literacy programs warrant evaluation to optimize the use of online information in infertility care.

## Conclusions

The Internet and media have become integral components of the information-seeking process for women experiencing infertility, offering rapid access to medical knowledge, peer experiences, and treatment-related information. In our study, online resources were perceived as useful for improving understanding of infertility and supporting decision-making. However, the heterogeneity, lack of personalization, and variable reliability of available content were frequently associated with increased anxiety and psychological burden.

These findings highlight the need for healthcare professionals to actively address patients’ online information-seeking behaviors during fertility consultations. By acknowledging patients’ use of digital resources, clarifying misconceptions, and directing them toward reliable, evidence-based, and culturally appropriate sources, clinicians can help mitigate misinformation-related stress and enhance patient empowerment. Integrating guided digital health literacy into infertility care may therefore improve patient experience, strengthen the physician-patient relationship, and support more informed and shared decision-making.
